# Development of PROTACS degrading KRAS and SOS1

**DOI:** 10.32604/or.2024.051653

**Published:** 2024-07-17

**Authors:** GERHARD HAMILTON, MARIE-THERESE EGGERSTORFER, SANDRA STICKLER

**Affiliations:** Institute of Pharmacology, Medical University of Vienna, Vienna, 1090, Austria

**Keywords:** Proteolysis-targeting chimeras (PROTACs), Kirsten rat sarcoma virus (KRAS), Son of sevenless 1 (SOS1), Von Hippel-Lindau, Cereblon

## Abstract

The Kirsten rat sarcoma virus—son of sevenless 1 (KRAS-SOS1) axis drives tumor growth preferentially in pancreatic, colon, and lung cancer. Now, KRAS G12C mutated tumors can be successfully treated with inhibitors that covalently block the cysteine of the switch II binding pocket of KRAS. However, the range of other KRAS mutations is not amenable to treatment and the G12C-directed agents Sotorasib and Adragrasib show a response rate of only approximately 40%, lasting for a mean period of 8 months. One approach to increase the efficacy of inhibitors is their inclusion into proteolysis-targeting chimeras (PROTACs), which degrade the proteins of interest and exhibit much higher antitumor activity through multiple cycles of activity. Accordingly, PROTACs have been developed based on KRAS- or SOS1-directed inhibitors coupled to either von Hippel-Lindau (VHL) or Cereblon (CRBN) ligands that invoke the proteasomal degradation. Several of these PROTACs show increased activity *in vitro* and *in vivo* compared to their cognate inhibitors but their toxicity in normal tissues is not clear. The CRBN PROTACs containing thalidomide derivatives cannot be tested in experimental animals. Resistance to such PROTACS arises through downregulation or inactivation of CRBN or factors of the functional VHL E3 ubiquitin ligase. Although highly active KRAS and SOS1 PROTACs have been formulated their clinical application remains difficult.

## Introduction

KRAS (Kirsten Rat Sarcoma virus) is the most important oncogene in pancreatic, colon, and lung cancer that quite recently can be targeted by inhibitors that covalently bind to the KRAS G12C mutant [1]. KRAS transmits the upstream activation of epidermal growth factor receptor (EGFR) and receptor tyrosine kinases (RTK) to the downstream RAF-MEK-ERK, PI3K-Akt-mTOR and RALGDS-RAL signaling cascades. Guanine nucleotide exchange factors (GEFs) and GTPase activating proteins (GAPs) cycle KRAS between the active GTP-bound and the inactive GDP-bound state. In detail, activated growth factor receptors interact with growth factor receptor-bound protein 2 (GRB2) that recruits the GEF Son of Sevenless 1 (SOS1) resulting in the displacement of GDP from KRAS. Oncogenic KRAS mutations are clustered in positions 12, 13, or 61 of the gene and lock KRAS in the GTP-bound active state leading to permanent overactivation and cell growth [2]. Two small molecules targeting KRAS-G12C, namely Amgen (AMG510-Sotorasib) and Mirati (MRTX849-Adagrasib) bind to the allosteric switch-II pocket and covalently link the cysteine residue and the acrylamide group of the KRAS G12C inhibitor, were recently approved for clinical use in docetaxel-pretreated lung cancer patients [3,4]. Sotorasib, the first-in-class KRAS inhibitor, yielded an overall response rate (ORR) of 41%, a progression-free survival (PFS) of 6.3 months, and proved superior to docetaxel. Adagrasib showed similar clinical efficacy with an ORR of 42.9% and a PFS of 6.5 months. Other KRAS mutations cannot be inhibited by drugs so far and for the G12C patients, the duration of the responses is approximately 8 months [5,6]. Chemoresistance develops early and the first KRAS inhibitor combination therapies are in clinical trials [7]. Modulators of upstream KRAS-interacting proteins, such as SOS1 and GRB2, are potential targets to enhance the inhibition of KRAS and the triggered signal transduction cascade. Another strategy is the replacement of inhibitors by proteolysis-targeting chimeras (PROTACs) that degrade the protein of interest (POI) in an event-based mode [8]. PROTACs are engineered from target-binding molecules and an E3 ubiquitin ligase ligand coupled by a chemical linker that brings the POI and the ligase in close contact, resulting in ubiquitylation and proteasomal degradation. The ligand must not inhibit the POI and reversible binding allows the PROTAC to recycle for further degradation events removing both the function and the scaffolding role of the POI.

### Proteolysis-targeting chimeras (PROTACs)

The conserved Ubiquitin-Proteasome System (UPS) removes both normal and misfolded cellular proteins through labeling with poly-ubiquitin chains to proteasomes for degradation [9,10]. PROTACs exert efficient degradation of the POIs compared to the corresponding small-molecule inhibitors with lower toxicity [11]. The activity of a PROTAC is not dependent on its affinity to the specific POI and low-affinity interaction directly correlated to the PROTAC’s affinity for the respective target and low-affinity interaction still permits an increased recycling [12,13]. Bond et al. reported the first KRAS G12C degrader, namely LC-2, that consisted of the covalent KRAS MRTX849 inhibitor coupled to a VHL E3 ubiquitin ligase ligand [14]. LC-2 demonstrated KRAS degradation and inhibition of the downstream MAPK signaling in several KRAS G12C mutant cell lines but the covalent interaction with the POI abolished recycling and limited the reactivity of this agent [15]. A reversible KRAS G12C PROTAC, termed YF-135, was reported which holds a cyanoacrylamide-based electrophile instead of the reactive acrylamide warhead exhibiting similar KRAS-directed activity compared to the LC2 compound [16].

Targeted protein degradation (TPD) modes are based on the UPS and catalyzation by E1 activating and E2 conjugating enzymes as well as E3 ligases and the proteasome. Currently, only several E3 ligase recruiters out of the more than 600 humans E3 ligases are used as E3 ligands for TPD [17,18]. In most cases, RING-type E3 ubiquitin ligase von Hippel-Lindau (VHL), cereblon (CRBN), and mouse double minute 2 (MDM2) were employed among others [19]. VHL and CRBN E3 ubiquitin ligases can be designed into PROTACs using AI-based structure prediction and screening of virtual docking models. The type, lengths, and rigidity of the coupling chemistry of the POIs and E3 ligands determine the efficacy of the PROTACs. These degraders afford multiple rounds of activity and eliminate both the enzymatic and scaffolding functions of the POI. In 2010, it was found that thalidomide and its analogs (pomalidomide and lenalidomide) directly bind to CRBN and CRBN-based degraders have been developed to treat human cancers [7]. CRBN-based E3 ligands have been selected for their superior oral bioavailability compared to other systems. Whereas ligands of CRBN are based on thalidomide derivatives, ligands for VHL E3 ligase comprise a hydroxylated proline within the recognition sequence of the hypoxia-inducible factor-1α (HIF-1α).

Approximately fifteen PROTAC degraders are in clinical Phase I-II trials with the ARV-471 and ARV-110 PROTACs from Arvinas, Inc. that are CRBN-containing PROTACs developed to target the androgen receptor (AR) and the estrogen receptor (ER), respectively. As the first PROTACs in human trials, ARV-110 and ARV-471 demonstrated satisfactory safety and efficacy results, proving the feasibility of the PROTAC therapy [20]. Of the fifteen clinically tested PROTACs, twelve contain CRBN E3 ligase, one has incorporated a VHL E3 ligase and the structure of two other PROTACs has not been revealed [21,22].

### KRAS-directed degrader

**Figure 1:**
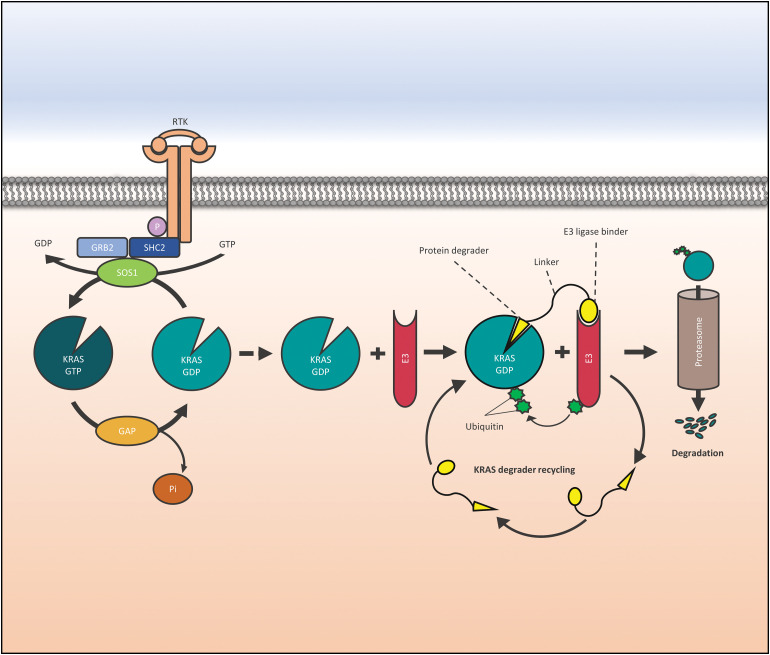
Function of a KRAS (Kirsten Rat Sarcoma virus) degrader. Activation of RTKs is transferred to KRAS via GRB2, SHC2, and SOS1, and its loading with GTP is followed by downstream activation of signaling cascades. In targeted protein degradation a protein degrader binds to KRAS and recruits an E3 ubiquitin ligase that directs the POI to the proteasome. Abbreviations are as follows: Receptor Tyrosine Kinase (RTK), factor receptor-bound protein 2 (GRB2), SHC Adaptor Protein 2 (SHC2), phosphate (P), Son of Sevenless (SOS1), Kirsten Rat Sarcoma virus (KRAS), Guanidine diphosphate (GDP), Guanidine triphosphate (GTP), GTPase activating proteins (GAP), E3 ligase (E3).

Early PROTACs recruiting VHL or cereblon E3 ubiquitin ligases to KRAS G12C contain covalently reacting KRAS binders [23,24]. However, KRAS binding by irreversible inhibition prevents the recycling of the degraders. Thus, classical KRAS inhibitors, KRAS-binding Designed Ankyrin Repeat Proteins (DARPins), or intracellular antibody fragments have been employed to formulate KRAS degraders [24]. In the following sections, examples of several KRAS degraders with distinct designs are presented (Fig. 1).

A KRAS-directed PROTAC was developed based on classical KRAS inhibitors designed by Boehringer Ingelheim (Boehringer Ingelheim, Ingelheim, Germany). Compound BI-2865 inhibited the proliferation of isogenic G12C, G12D, or G12V mutant KRAS cells with a mean IC50 of approximately 140 nM [23]. An analog of BI-2865, namely BI-2493, binds the inactive state of KRAS, lacks activity against NRAS and HRAS, and is suited for *in vivo* administration. The incorporation of such inhibitors resulted in the panKRAS-directed ACBI3 PROTAC exhibiting VHL engagement and potent E3-ligase-dependent cellular degradation. Tests in 240 cancer cell lines revealed a broad and selective anticancer activity of ACBI3 for cell lines bearing KRAS mutations *vs.* WT KRAS cell lines (IC50 = 478 nM *vs*. 8.3 μM, respectively). PROTAC-mediated KRAS degradation revealed a 10-fold higher potency compared to inhibition using the cognate KRAS inhibitor and ACBI3 has the advantage to degrade several oncogenic KRAS mutants.

Two RAS degraders have been developed linking protein macromolecules to specific E3 ubiquitin ligases [24]. In detail, a KRAS-specific Designed Ankyrin Repeat Protein (DARPin) fused to the VHL E3 ubiquitin ligase and a pan-RAS intracellular single domain antibody (iDAb) coupled to the U-BOX of the C-terminus of Hsc70-interacting protein (CHIP) E3 ligase were designed as KRAS degraders [25–27]. The KRAS-specific DARPin degrader disintegrated both mutant and wildtype KRAS but inhibited only mutant KRAS cells *in vitro* and *in vivo*. The pan-RAS degrader directed by an antibody fragment reduced the proliferation of cancer cells independent of the specific RAS mutation.

### KRAS G12D degrader

A series of KRAS G12D PROTACs have been developed by coupling MRTX1133 analogs and VHL to target the most frequent KRAS mutation [28,29]. Variations of the linker group and the KRAS inhibitor MRTX1133 demonstrated the dependency of the PROTAC activity on the cell membrane permeability. The final PROTAC 80 demonstrated the potent decay of KRAS G12D mediated by a VHL-dependent proteasomal process. Furthermore, PROTAC 80 inhibited the proliferation of a panel of KRAS G12D mutant cancer cell lines, showed good bioavailability in mice, and suppressed the growth in a KRAS G12D-positive AsPC-1 xenograft model. The development of KRAS degraders for clinical application should check for proper pharmacokinetics and toxicity as far as these properties can be assessed in preclinical models [23,30].

### SOS1-directed degrader

SOS1 drives KRAS in its function as GEF and constitutes a therapeutic target that is independent of the specific KRAS mutation [31]. Thus, SOS1 inhibitors and degraders represent potential pan-KRAS agents that may be active in combination with the approved KRAS G12C inhibitors and help to retard acquired resistance mechanisms. Through the elimination of the SOS1-KRAS protein-protein interaction (PPI), these agents aim to impair the downstream signaling cascades and suppress cancer cell growth in KRAS-driven cancers, for example, colon cancer (CRC) [32,33]. In preclinical studies, SOS1 inhibitors showed an additive effect in combination with KRAS G12C inhibitors [34]. The SOS1 degraders were developed as PROTACs making use of the 3D models of SOS1 and CRBN aiming at improved selectivity and efficacy [35]. In general, the 6- and 7-methoxy groups of the quinazoline core of the basic structure of SOS1 inhibitors are suitable for coupling lenalidomide as CRBN ligand and were employed to formulate degrader P7 [36].

This SOS1 degrader P7 exhibited anticancer activity against CRC patient-derived organoids (PDO) growth with a 5-times increased activity compared to the SOS1 inhibitor BI-3406. This may be in part due to the removal of the scaffolding function of SOS1 in addition to the cycles of degradation [37]. The P7 SOS1 involves the BAY-293 inhibitor coupled to lenalidomide via a linker optimized by docking of BI68BS-bound SOS1 with lenalidomide-loaded CRBN. This SOS1 PROTAC degrader differs from the First-in-Class agonist-based SOS1 degrader 9d by the VUBI-1 agonist and the use of a VHL E3 ligase [38]. The CRBN is an E3 ligase regarded to provide better oral availability in contrast to the VHL ligase. Furthermore, the short linker offers a rigid PROTAC structure that efficiently targets mutant KRAS in CRC [39].

The SOS1-directed PROTAC 9d contains the VUBI-1 SOS1 agonist that kills tumor cells by a downregulation of the MAPPKs in response to SOS1 overactivation [40]. With a KD value of 44 nM, a solvent-exposed piperazine substituent of the agonist provided an attachment site for the E3 ligase ligand [40]. PROTACs were designed by tethering the VUBI-1 solvent-exposed piperazine substituent to the VHL ligand 8 with an 8-carbon linker. The resulting compound 9d showed a SOS1 removal of 56%–92% Dmax at concentrations of 0.1 and 1 μM, respectively. Importantly, the KRAS protein remained unchanged after 24 h treatment with 9d and the inhibition of cancer cells has been found in all types of distinct KRAS mutations. Furthermore, 9d suppressed the tumor growth of NCI-H358 xenografts with high efficacy with no major toxicity [40].

### Clinical administration of targeted protein degraders

TPD employs relatively small and stable drugs for the depletion of POIs giving much higher activity compared to the cognate KRAS or SOS1 binders [41,42]. PROTACs cycle through multiple rounds of degradation functioning at a sub-stoichiometric agent-target ratio. So far, the design of the current PROTACs resides on VHL and CRBN complexes as ubiquitin ligases. However, the human repertoire of over 600 ubiquitin ligases is expected to comprise enzymes with superior properties, such as tissue-restricted expression, cancer cell specificity or substrate recognition characteristics [11,21]. Today, most PROTACs use established and highly specific ligands for POIs, such as compounds directed to kinases, nuclear hormone receptors, and bromodomain-containing proteins that may be widened to further proteins and as yet undruggable targets [43]. In plasma, free fractions of small drugs are supposed to reach their target site but PROTACs often exhibit high lipophilicity that leaves minute amounts of free drug levels and impede drug determinations [44,45]. In most cases, the abundance of the POIs is valid as a pharmacological biomarker of the efficacy. Development of PROTACs usually involves the selection of suitable degraders in preclinical *in vivo* models. CRBN-based PROTACs have been shown to effect *in vivo* degradation in rodents without major side effects as have the Arvinas CBRN-based PROTACs in human trials. However, PROTACs may have molecular glue side been shown to effect and since human and murine CRBN are different assessment of their safety would likely need to be performed in monkeys [46].

PROTACs derived from kinase inhibitors have been demonstrated to show higher selectivity compared with inhibition probably due to restricted formation of ternary complexes with the targeted POIs in contrast to off-targets interaction [47]. Induced proximity of proteins that interact with the POI may lead to degradation by the so-called “bystander effect” [48,49]. The response of patients to degraders may be assessed in the clinical setting by proteomics of tissue biopsies [50]. These findings demonstrate that PROTACs for increasing directed to kinases have advantages but removal of the whole kinase proteins and of possibly bystanders need to be checked extensively in preclinical models ahead of clinical application. PROTACs are expected to play an important role in future therapies but need further major efforts to secure efficacy, selectivity, and safety for patient administration.

### Resistance to PROTACS

As for other chemotherapeutics, chemoresistance against PROTACs has already been reported in clinical trials, in particular for VHL- and CRBN-based agents and cancer cells during long term application [51]. For example, a poor response to the thalidomide-based immunomodulatory drugs (IMiDs) in multiple myeloma (MM) patients has been linked to their low expression of CRBN although higher expression of CRBN is usually found in cancer cell lines from hematological tumors compared to solid cancers [52,53]. According to data from the Human Protein Atlas almost all prostate cancer patients express CRBN at high levels, which is true for only 10% lung cancer patients [53]. Lower expression of CRBN E3 ubiquitin ligase components, such as DDB1 or CUL4, can also impair the efficacy of IMiD-PROTACs. Mutations in CRBN that disturb interactions with IMiD such as a nonsense mutation eliminating IMiDs binding domain will lead to chemoresistance.

The cellular dependence on CRBN has been tested for 1070 haploid cell lines via genomic knockout and reported to be non-essential in contrast to VHL that proved essential in 935 of the cell lines [54,55]. In CRBN-based dBET6 most disruptive alterations affected CRBN. In contrast, for cells resistant to the VHL-based ARV-771, genomic alterations were rare in VHL but frequent in other components of the CRL2VHL complex with most alterations holding heterozygous missense point mutations. Accordingly, one third of patients resistant to pomalidomide therapy express distinct types of CRBN alterations [56,57].

Different POI ligands, various E3 ubiquitin ligases and the selection of linkers dictate the selective degradation of similar members among protein families [58,59]. The development of PROTACs for efficient clinical application must overcome the inherent low cellular permeability and oral bioavailability [60]. The formation of productive ternary complexes is preceded by the generation of competing and ineffective binary complexes of PROTACs with POI or E3 ligases at high degrader concentrations for PROTACs binding, described as “hook effect” [61]. A largely open question is the occurrence of the side effects and toxicity of PROTACs that may be on-target as well as off-target [8,62]. Unacceptable toxicity may arise from the complete degradation of POIs due to extensive cycles of degradation, not only in target cells but likewise in normal tissues [63,64]. For instance, BET bromodomain proteins are impeded by their inhibitors, but the complete degradation of the bromodomain BRD2 and BRD4 family members is lethal for the cells. The introduction of tumor-specific E3 ligases could avoid uncontrolled protein degradation and toxicity on normal cells [64,65]. Furthermore, evolution of alternative E3 ligase binders may improve the physicochemical characteristics of PROTACs [66]. Ultimately, the tissue or disease specificity of the PROTACs is determined by the degree of expression of the required E3 ligases and the cognate POIs in target cells [64,65,67]. High expression of CRBN or VHL E3 ligases may engage PROTACs for increasing cytotoxicity and side effects, thus preventing clinical application, such as for the PROTACs directed to CDK9 or AURKA, respectively [68,69]. Knockout of mutant KRAS may be compensated in cancer cells securing further survival [70]. Thus, depletion of KRAS may be possible without severe side effects but contribute to a potential resistance mechanism to RAS PROTACs.

Novel approaches have been published to deliver PROTACs to cancer cells. For example, the folate receptor α (FOLR1) is overexpressed in malignant cells and the folate-caged is a well-defined target for drug delivery [71]. Liu et al. developed a folate-caged formulation BRD-PROTAC (ARV-771) that has been efficiently transferred intracellularily and degraded BRDs via cleavage by endogenous hydrolase in FOLR1-positive cancer cells [72]. Furthermore, the selectivity of PROTACS could be enhanced when formulated into antibody-PROTAC conjugates (Ab-PROTACs) or as antibody-based PROTACs (AbTACs) [73]. To date, there are still no reproducible rules and principles to improve the pharmacokinetics and efficacy of PROTACs and their development has to be guided by laborious screening *in vitro* and *in vivo*.

## Conclusion

Targeting mutated KRAS has the potential to improve the prognosis of approximately 7% of all cancer cases. The approved inhibitors of KRAS G12C mutated cancers procure responses in less than half of the patients, with less than 1-year duration but extended disease control. Combination with SOS1 GEF inhibitors is expected to increase the clinical activity of KRAS inhibitors. Higher efficacy and coverage of non-G12C mutated KRAS may be achieved by designing the inhibitors into PROTACS. An increasing series of KRAS- or SOS1-directed PROTACs has been reported based on CRBN or VHL as E3 ubiquitin ligases. These PROTACs exhibit higher activity *in vitro* and in experimental animal models compared to their cognate inhibitors, covering most other KRAS mutations. First-line therapy to abolish KRAS-SOS1 signaling events by degraders may still require combinations with other agents for maximal efficacy. Feedback activation of the KRAS/SOS1 circuit or compensatory mechanisms involving HRAS and NRAS may result in chemoresistance. The real difficulty regarding these PROTACs is the formulation for their clinical use and the testing of the possible target-specificity and other side effects. Although first CRBN-based PROTACs are in clinical studies for breast and prostate cancer with sufficient safety, this could be different for other POIs. The relatively high molecular weight, high polarity, low solubility, and low permeability of the PROTACs pose problems for the oral bioavailability of the compounds. The effects of partial or complete degradation of mutated KRAS or SOS1 in cancer cells and bystander normal tissues are not clear. By using tissue-specific or tumor-selective E3 ligase ligands PROTACs may afford tumor-selective degradation of target proteins. Resistance to PROTACs has been described and mostly involves downregulation or mutations of CRBN or components of the VHL ligase. CRBN-depleted cells become resistant to lenalidomide and pomalidomide but not to other drugs. However, PROTACs offer a range of possibilities for improved cancer therapy that reaches beyond the current inhibitors.

## Data Availability

Available on reasonable request.
